# How to Keep Cool

**Published:** 1881-10

**Authors:** 


### HOW TO KEEP COOL.

On going once into the Medical Museum in Edinburgh, on a summer's day, we felt chilly, and on looking at the Thermometer we found it at sixty-eight, while out of doors it was oppressively warm. Sixty-eight degrees in summer there, is quite cool enough for a sitting apartment; but if you go into a room of that temperature in mid-winter, a feeling of suffocation, of oppressiveness, comes over you. The noon of a day whose morning is sixty-eight will give over ninety in the sun. If on getting up in the morning, every window and door of "*a floor*" are thrown open and thus remain until about sun up, and are then closed, shutters and all, it will be nearly night before the thermometer is materially raised, and persons coming into our office, often exclaim, "how delightfully cool your office is, how do you manage it." If we close our doors in mid-winter to keep the warmth in, may we not do the same thing in summer to keep it out?

